# Current Concepts of Vitiligo Immunopathogenesis

**DOI:** 10.3390/biomedicines10071639

**Published:** 2022-07-08

**Authors:** Nika Hlača, Tina Žagar, Marija Kaštelan, Ines Brajac, Larisa Prpić-Massari

**Affiliations:** Department of Dermatovenerology, Medical Faculty, University of Rijeka, Clinical Hospital Center Rijeka, Krešimirova 42, 51000 Rijeka, Croatia; tinazagar89@gmail.com (T.Ž.); marija.kastelan@uniri.hr (M.K.); ines.brajac@medri.uniri.hr (I.B.)

**Keywords:** autoimmune diseases, dendritic cells, keratinocytes, melanocytes, memory T cells, natural killer cells, oxidative stress, Th1 cells, vitiligo

## Abstract

Vitiligo is an acquired immune-mediated disorder of pigmentation clinically characterized by well-defined depigmented or chalk-white macules and patches on the skin. The prevalence of vitiligo varies by geographical area, affecting 0.5% to 2% of the population. The disease imposes a significant psychological burden due to its major impact on patients’ social and emotional aspects of life. Given its autoimmune background, vitiligo is frequently associated with other autoimmune diseases or immune-mediated diseases. Vitiligo is a multifaceted disorder that involves both genetic predisposition and environmental triggers. In recent years, major predisposing genetic loci for the development of vitiligo have been discovered. The current findings emphasize the critical role of immune cells and their mediators in the immunopathogenesis of vitiligo. Oxidative-stress-mediated activation of innate immunity cells such as dendritic cells, natural killer, and ILC-1 cells is thought to be a key event in the early onset of vitiligo. Innate immunity cells serve as a bridge to adaptive immunity cells including T helper 1 cells, cytotoxic T cells and resident memory T cells. IFN-γ is the primary cytokine mediator that activates the JAK/STAT pathway, causing keratinocytes to produce the key chemokines CXCL9 and CXCL10. Complex interactions between immune and non-immune cells finally result in apoptosis of melanocytes. This paper summarizes current knowledge on the etiological and genetic factors that contribute to vitiligo, with a focus on immunopathogenesis and the key cellular and cytokine players in the disease’s inflammatory pathways.

## 1. Introduction

Vitiligo is an immune-mediated disorder of pigmentation clinically characterized by well-defined depigmented or chalk-white macules and patches on the skin. The prevalence of vitiligo varies depending on the geographical area, from 0.5% to 2% of the population, equally affecting adults and children [1,2]. Some geographical regions have a higher prevalence of vitiligo; for example, India (8.8%) and Mexico (2.6–4%) are countries with the highest reported incidence of vitiligo [2,3]. According to a recent meta-analysis on the prevalence of vitiligo, the variability in epidemiological data could be due to differences in skin types and ethnic groups across areas, as well as environmental and genetic factors that may contribute to the disparity in prevalence [3]. Vitiligo may appear at any age, with a peak incidence in the second and third decades of life. Nearly one-third of vitiligo patients are children under ten years of age [4]. Although patients can often associate vitiligo onset and the emergence of new lesions with certain life events such as emotional stress, physical trauma, sunburn, and pregnancy, there is only clear evidence of a link between vitiligo and the isomorphic response, also known as the Koebner phenomenon [5]. At the beginning or during the active spread of the lesion, vitiligo may appear ill-defined and hypopigmented [1]. Vitiligo may be localized anywhere on the body, although there is a predilection for the face, particularly periorificial region, genitals, and hands [4]. Still, the lesions may become noticeable only after examination under a Wood lamp in people with fair skin. Halo nevus (HN) has also been associated with vitiligo and may precede the development of generalized vitiligo [6]. HN is a melanocyte nevus surrounded by a sharply demarcated zone of depigmentation. The central, melanocyte portion of the HN may partially or completely disappear, whereas the associated halo depigmentation may be maintained or even expanded. HN can be single or multiple, and multiple HNs occur in 20–50% of patients. Histological features of HN show great similarities with vitiligo. Moreover, 20% of patients with HN also have vitiligo [7,8]. Given its autoimmune background, vitiligo is often linked to other autoimmune diseases or immune-mediated diseases [9,10,11,12]. Patients with comorbid autoimmune disorders have a higher incidence of the generalized form of the disease.

According to the revised classification proposed in 2012 by Vitiligo Global Issues Consensus Conferences, vitiligo is classified into three categories: non-segmental vitiligo (NSV), segmental vitiligo (SV), and undetermined/unclassified group [13]. Differentiating SV from other subtypes is vital because of its prognostic implications. The term non-segmental vitiligo is suggested for all NSV forms of vitiligo. It is further divided into a generalized (GV), acral or acrofacial, mucosal, localized, universal, and mixed pattern based upon the distribution of skin lesions. Generalized and acral or acrofacial subtypes of vitiligo are the most common. Segmental vitiligo is a rare type of disease. SV typically has a dermatome or quasi-dermatome pattern, begins in childhood, rarely spreads beyond the affected dermatome, and may be monosegmental, bisegmental, or plurisegmental. The immunopathogenetical mechanisms of NSV and SV also differ. In NSV, immune activation is systemic, whereas in SV, a local cytotoxic reaction is involved. Furthermore, unlike in SV, NSV patients have fewer regulatory T cells, making them more susceptible to other systemic autoimmune diseases [14]. The following manuscript will focus on NSV, further referred to as vitiligo.

## 2. Genetic Factors in the Development of Vitiligo

Vitiligo is multifaceted disorder involving simultaneous contribution of multiple genetic risk factors and environmental triggers [11,15,16]. In recent years, genome-wide association study (GWAS) has shed light on the genetic basis of vitiligo [11]. The incidence of vitiligo within certain families is suggestive of the genetic background of the disease, although the genetic factor is not absolute [15]. Genetic studies suggest a multifactorial, polygenic inheritance pattern, with significant genetic heterogeneity in different ethnic groups [17,18]. It is estimated that about 15–25% of patients have at least one first-degree relative affected by vitiligo [19]. Additionally, monozygotic twins’ studies in European-derivated whites found a concordance rate of 23%, indicating that different environmental or non-genetic factors contribute to development of vitiligo [18]. Over the past decades, many genes and genomic regions for vitiligo susceptibility have been implied through linkage analysis and candidate gene studies [11]. So far, GWAS has identified 50 distinct vitiligo susceptibility loci, mostly in people of European and Chinese descent. Approximately half of the identified vitiligo susceptibility genes encode proteins important in regulation of immune system and apoptosis, whereas several encode melanocyte-specific proteins. Interestingly, about half of the vitiligo susceptibility genes have also been recognized in genetic studies of other autoimmune diseases and autoinflammatory disorders connected to vitiligo, including thyroid disease, type 1 diabetes (T1D), and rheumatoid arthritis, suggesting a shared autoimmune predisposition [20].

The human leukocyte antigen (HLA) harbors many genes important for immune system [21]. In Caucasians, the peaks for vitiligo susceptibility in the MHC region were detected in the class I gene region between HLA-A and HCG9 and the class II region between HLA-DRB1 and HLA-DQA1 [22,23,24,25,26]. Interestingly, psoriasis and vitiligo, share a common genetic locus in the MHC region, suggesting they may even share some similar molecular mechanisms [27,28]. Kemp et al. reported the first vitiligo non-MHC candidate gene associated with CTLA4, which encodes a T-cell co-receptor involved in the regulation of T-cell activation [29,30]. Another non-MHC candidate gene was PTPN22, which encodes LYP protein tyrosine phosphatase, a regulator of the activation and development of T-cells. GWAS studies of European whites replicated these associations, but they were not applicable to other populations [31,32]. Along with HLA class II, CTLA4 and PTPN22 are likely two of the genes that underlie the epidemiologic association of vitiligo with other autoimmune diseases in European-derived whites [16].

Another immune regulatory gene reported by GWAS is CD80. CD80 encodes a surface protein on activated B-cells, monocytes, and dendritic cells that co-stimulates T cell priming [33,34,35]. Interestingly, flow cytometric analysis has found a significantly increased percentage of CD80+ monocytes in the vitiligo group compared with the controls, which may indicate alteration of monocytes function in vitiligo [36]. TICAM1, also known as TIR domain-containing adaptor-inducing IFN-b, which encodes Toll-like receptor adaptor molecule 1, mediates the innate immune response to viral pathogens, might act as a viral factor in the pathogenesis of vitiligo [37,38]. IKZF4 is an additional biological candidate gene for GV identified by two GWASs [33,39,40]. It is also associated with T1D and alopecia areata [41,42]. IL2RA, which encodes the interleukin-2-receptor alpha chain on chromosome 10p15.1,8 is another gene strongly associated with GV [22]. Elevated interleukin-2-receptor serum levels in GV patients indicate T-cell activation [43].

Several genes that are only expressed in melanocytes and are involved in melanocyte function have been identified as vitiligo susceptibility loci, in addition to immune regulating genes [44,45]. The aforementioned genes encode enzymes or proteins that have been identified as vitiligo autoantigens. Tyrosinase is an enzyme of the melanocyte that catalyzes the rate limiting steps of melanin biosynthesis. Tyrosinase encoded by TYR represents a major autoantigen in generalized vitiligo [22,46]. Biological interaction between HLA-A and TYR shows an inverse relationship between susceptibility to vitiligo versus malignant melanoma (MM) [12]. This suggests that vitiligo may be caused by dysregulation of immune surveillance mechanisms which are critical against MM [47]. Interestingly, it unexpectedly turned out that most vitiligo susceptibility genes that encode proteins involved in melanocyte function have also been associated with melanoma but with opposite effects.

PMEL gene encodes a melanocyte-specific type I transmembrane glycoprotein. The encoded protein plays an essential role in the structural organization of premelanosomes [48]. Transcriptome analysis of vitiligo lesional skin shows decreased PMEL expression compared to vitiligo perilesional unaffected skin [39]. MC1R encodes the receptor protein for melanocyte stimulating hormone (MSH). It is a minor vitiligo autoantigen that regulates melanogenesis and is associated with MM as well as skin and hair color [49]. Further studies should be conducted to clarify the MC1R gene’s ability to protect against vitiligo [50]. Genes involved in gene repression, apoptosis, and cell survival are another group of genes that may play a role in the progression of vitiligo [33]. CASP7, for example, encodes a member of the cysteine-aspartic acid protease family (caspase) and is essential in the execution phase of cell apoptosis and inflammation [51].

GWAS in whites of European ancestry recognized a genetic association between vitiligo and GZMB [22,33]. GZMB is caspase alike serine protease that mediates two processes that terminate the immune response [52,53]. Apart from GV, GZMB has been linked to juvenile idiopathic arthritis and Bechet’s disease [54,55]. RERE is another gene identified in association with vitiligo, which encodes proteins involved in cellular apoptosis [22,33]. Furthermore, the NALP1 gene on chromosome 17p13, which encodes NACHT, belongs to the regulators of the innate immune system and is also a gene associated with vitiligo and other autoimmune diseases [9]. The majority of genes associated with immunoregulatory proteins are also linked to other autoimmune diseases related to vitiligo.

Although many specific biological mechanisms resulting from genetic factors remain unknown, it is clear that vitiligo is an immune-mediated disease involving a complex relationship between immune system programming and function, aspects of melanocyte, and immune dysregulation.

## 3. Current Concepts in Immunopathogenesis of Vitiligo

**Figure 1:**
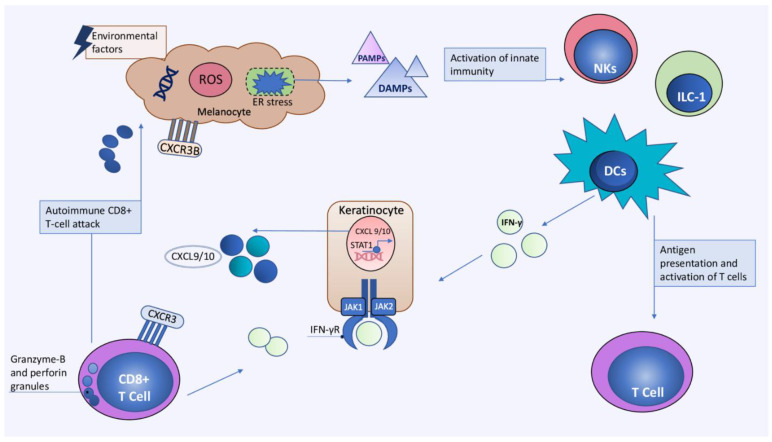
Main effector cells and signaling pathways in the initial stage of vitiligo. Vitiligo immunopathogenesis is characterized by a complex inflammatory cascade that is initiated by innate immune cells (DCs, NK cells, and ILC-1 cells) which recognize signals from stressed melanocytes (DAMPs and PAMPs). Following activation, innate immune cells begin to produce IFN-γ, which activates JAK/STAT pathway and causes keratinocytes to produce the chemokines CXCL9 and CXCL10. These chemokines are in responsible for recruiting CD8+ cells to the skin via the CXCR3 receptor, where the effector phase of the anti-melanocyte immune response occurs. CD8+ lymphocytes are an additional source of IFN-γ as well as the cytolytic molecules perforin and granzyme-B, capable of inducing melanocyte apoptosis. Furthermore, CXCL10 can induce melanocyte apoptosis via CXCR3B. CXCL9, CXC chemokine ligand 9; CXCL10, CXC chemokine ligand 10; CXCR3, chemokine receptor type 3; DAMP, damage-associated molecular pattern; DCs, dendritic cells; ER; endoplasmic reticulum; IFN-γ, interferon-γ; ILC-1, group 1 innate lymphoid cell; JAK, Janus kinase; NKs, natural killer cells; PAMP, pathogen-associated molecular patterns; ROS, reactive oxygen species; STAT1, signal transducer and activator of transcription 1.

Similar to other immune-mediated skin diseases, vitiligo is a multifactorial disease caused by complex interactions between genetic and environmental factors [56,57,58]. The current data highlight the central role of immune cells and their mediators in the immunopathogenesis of vitiligo. The key immune cells include T helper 1 cells, cytotoxic T cells, regulatory and resident memory T cells, as well as dendritic cells (DCs), natural killer (NK) and ILC-1 cells. Moreover, key mediators such as interferon-γ (IFN-γ), C-X-C chemokine ligand 9 (CXCL9) and 10 (CXCL10), as well as interleukin-15 (IL-15) are also implicated in etiology of vitiligo [4,56,59]. Additionally, non-immune cells such as keratinocytes, fibroblasts, and stem cells contribute to vitiligo pathogenesis. However, the exact events leading to the development of vitiligo are still incompletely understood. Oxidative stress is currently accepted as the initial event leading to the activation of innate immunity in the earliest stages of vitiligo [60]. Genetic factors, together with environmental factors induce melanocyte and keratinocyte stress, represented by endoplasmic reticulum (ER) stress. Following exposure to stressful triggers, melanocytes release reactive oxygen species (ROS) which have the potential to alter cellular DNA, proteins, and lipids. This leads to the production of several damage-associated molecular patterns (DAMPs) such as melanocyte-specific antigens, miRNAs, and heat shock proteins (HSP) [60,61,62]. Inducible HSP protein 70 (iHSP70) is particularly important in bridging native and adaptive immunity since it can stimulate the transformation of dendritic cells into efficient antigen-presenting cells [63]. Dendritic cells (DC) present melanocyte-derived peptides to T cells leading to their differentiation and production of interferon-γ (IFN-γ) [61,63]. IFN-γ is the key cytokine mediator that leads to the phosphorylation of the transcription factor STAT1 by Janus kinases (JAK) 1 and 2. Phosphorylated STAT1 homodimerizes and enters the nucleus, where IFN-γ-dependent genes, such as CXCL9 and CXCL10 (C-X-C chemokine ligand 10), are transcribed [64,65]. CXCL9 and CXCL10 are responsible for the recruitment of autoreactive CD8+ T cells to the skin via their shared C-X-C chemokine receptor 3 (CXCR3) [65]. Impairment of regulatory T cells further enhances the CD8+ anti-melanocyte response. Finally, melanocyte apoptosis is mediated by granzyme B and perforin released from CD8+ T lymphocytes or alternatively via chemokine induction of the CXCR3B receptor on melanocytes [66,67,68] (Figure 1).

### 3.1. The Role of Oxidative Stress

The skin functions as a barrier organ providing protection against external stressors. UV radiation, microorganisms, and oxidizing chemicals are examples of exogenous stimuli that target melanocytes and cause ROS production [60,61,69,70]. Thus, melanocytes employ several mechanisms to maintain redox homeostasis. Antioxidants, including superoxide dismutase, catalase, glutathione, vitamin C, and vitamin E may counteract the effects of ROS. Recent studies, however, have shown an imbalance in antioxidants, with higher levels of superoxide dismutase, lower levels of catalase, and increased lipid peroxidation in patients with vitiligo [60,61]. Additionally, elevated levels of ROS are found in both lesional and non-lesional skin of patients with vitiligo [71], further emphasizing their decreased ability to resist ROS [72]. Apart from exogenous sources of ROS, there are also endogenous ROS that mostly result from melanin synthesis [61,70]. However, vitiligo melanocytes exhibit several structural defects that impair their ability to resist oxidative stress, including endoplasmic reticulum dilatation, mitochondrial dysfunction, and abnormal melanosome structure [69]. Dilatation of the ER occurs because of the buildup of misfolded proteins, perpetuating ER stress and oxidative stress [73,74]. Additional instability of tyrosinase-related protein-1 (TYRP1), which is required for melanogenesis, leads to accumulation of melanin intermediates in patients with vitiligo [75,76,77]. Melanin intermediates, together with other stressors such as heat shock or ROS, disrupt the protein folding process in the ER, activating the unfolded protein response (UPR) and integrated stress response (ISR) [78,79]. UPR is one of the key factors driving the formation of neoantigens, which have the potential to activate innate immunity and eventually adaptive immunity against melanocytes [73,79]. The role of UPR has already been established in several autoimmune diseases, with type 1 diabetes (T1D) being one of the most studied [80,81]. In the skin and serum of patients with vitiligo, UPR promotes the production of the chemokines IL-6 and IL-8 through the transcription factor X-box-binding protein 1 (XBP1) [82]. Polymorphisms in UPR-linked genes, such as XBP1 and UVRAG, are associated with an increased risk of vitiligo [78,83]. The impaired function of the nuclear factor erythroid 2-related factor 2 (NRF2) antioxidant pathway further contributes to dysfunctional UPR activation in vitiligo. NRF2 is a nuclear factor employed in various antioxidant systems, such as the glutathione and thioredoxin antioxidant systems [84,85]. Since vitiligo melanocytes lack this protective antioxidant mechanism, they are vulnerable to the depigmenting chemical phenol monobenzone [86,87]. Therefore, targeting the NRF2 pathway may be a possible therapeutic option for patients with vitiligo [88,89].

Additional potential players in mediating oxidative stress response are small non-coding RNAs (ncRNAs), especially microRNAs (miRNAs) [90]. miRNAs regulate post-transcriptional gene expression by inducing degradation or inhibiting translation of mRNA to proteins [91]. Through targeting complex gene pathways, miRNA may provide a link to ER stress and appearance of vitiligo [92]. Previous studies have demonstrated alterations in multiple miRNAs expression affecting both skin and blood of patients with NSV [93]. MicroRNA421 (miR421) is one of the miRNAs associated with oxidative stress response in vitiligo. ER stress increases the expression of miR421 and several ER stress related proteins, whereas miR421 inhibition reduces the expression of ER stress related proteins and decreases melanocyte apoptosis. As a result, miR421 may be a promising therapeutic target for vitiligo [91]. The expression of miR-25 additionally increases in response to oxidative stress which causes degeneration of melanocytes but also alters keratinocytes’ protective paracrine function, and thus may be worth further investigation as a potential therapeutic target [94]. In contrast, miR-211, which is involved in mitochondrial metabolism and oxidative phosphorylation, is downregulated in vitiligo and may partly explain the increased ROS production and impaired respiratory responses seen in the disease [95]. Further research into various miRNAs and their target genes could provide useful insight into the molecular basis of vitiligo and even lead to the development of therapeutic targets or disease biomarkers.

Studies on melanocyte detachment mechanisms have recently emerged as an important factor in melanocyte loss. Notably, when IFN-γ and TNF-α are present, keratinocytes release matrix metalloproteinase (MMP)-9, which causes melanocyte detachment from the basal membrane by targeting E-cadherin. E-cadherin is a key protein involved in melanocyte adhesion. Interestingly, E-cadherin levels are lower in vitiligo patients’ perilesional skin, whereas MMP-9 levels are higher in the skin and sera of patients with vitiligo [96,97]. Another protein with similar function is the melanoma inhibitory activity (MIA) protein, which targets alpha5beta1 integrins (a5b1ints) and can induce melanocyte detachment. The presence of MIA in vitiligo patients has already been established [98]. A recent study on the vitiligo mouse model revealed that the introduction of MIA resulted in the formation of a depigmentation zone that histologically corresponded to vitiligo. However, because there were no immune cells in this vitiligo model, it is unclear how MIA is related to oxidative stress or immune-mediated vitiligo mechanisms. Perhaps, IFN-γ or oxidative stress may be responsible for inducing melanocyte senescence followed by release of MIA which is yet to be investigated [99].

Despite the lack of specific mechanisms underlying the link between innate immunity and oxidative stress, oxidative stress provides a plausible explanation for the onset of vitiligo and subsequent activation of innate immunity.

### 3.2. The Role of Innate Immunity

Innate immunity is frequently referred to as a bridge between oxidative stress and adaptive immunity [100]. That is due to the ability of innate immune cells to recognize stressed or damaged cells via pattern recognition receptors (PRRs) [45,59]. Among the cells of innate immunity, NK cells and DCs are the most studied in vitiligo. Previous studies have found systemic and local increase in the function and expression of NK cells [66,67,101]. In addition to NK cells, DCs are additional players in the innate immunity through recognition of DAMPs released by stressed melanocytes. Following DC maturation, melanocyte-associated antigens are presented to T cells in lymphoid tissues, resulting in a T-cell mediated anti-melanocyte response [63,82,102]. In addition, increased levels of innate immunity cytokines including interleukin (IL)-1α, IL-1β, IL-6, IL-8, IL-12, IL-15 and tumor necrosis factor (TNF)-α have been detected in the sera of patients with vitiligo [103]. Evidence on the role of innate immunity has furthermore been established from genetic studies showing upregulation of genes involved in innate immunity in the lesional and non-lesional skin of vitiligo patients [101]. Additionally, genes related to innate immunity including NALP1, TICAM1, IFIH1 are recognized as vitiligo susceptibility genes [9,15,100,104,105]. As suggested above, the innate immunity pathways are certainly implicated in the onset and progression of vitiligo.

#### 3.2.1. The Role of DAMPs and PAMPs

The accumulation of ROS in melanocytes under oxidative stress has the potential for carbonylation and oxidation of melanocyte proteins, leading to the formation of neoantigens or DAMPs [106]. Pattern recognition receptors (PRRs) such as Toll-like receptors (TLR), RIG-I-like receptors (RLRs), and nucleotide oligomerization domain (NOD)-like receptors (NLRs) allow cells of the innate immune system to recognize stressed or damaged melanocytes [107,108]. HSP70 is one of the most recognized DAMPs in vitiligo, correlating well with disease activity [63]. Exposure to certain chemicals such as 4-tertiary butyl-phenol (4-TBP) may induce the production of HSP70 by vitiligo melanocytes [63,109]. Vitiligo melanocytes consequently express tumor necrosis factor-related apoptosis-inducing ligand (TRAIL) on their surface, leading to the infiltration of DCs into the perilesional skin of patients with vitiligo. Recently, a study on vitiligo in a Sinclair swine model found that the altered version of iHSP70, that is, HSP70iQ435A may induce the repigmentation of vitiligo lesions, thus making it a potential therapeutic target [110]. Another important DAMP is the high-mobility group protein B1 (HMGB1), which can mediate oxidative stress response and activation of DCs [106]. HMBG1 is released from both activated immune or non-immune cells such as melanocytes and keratinocytes, and levels of HMBG1 are increased in sera and lesional skin of patients with active vitiligo [85,111]. The key mediators of HMGB1, CXCL16, and IL-8, produced by keratinocytes lead to CD8+ T-cell chemotaxis and infiltration of the perilesional skin [111,112]. In addition to HSP70 and HMGB1, calreticulin (CRT) is also a messenger of the melanocyte oxidative response. CRT is an ER protein employed in Ca^2+^ homeostasis and signaling. CRT can enhance the immunogenicity of melanocytes directly by increasing its expression on their surface or indirectly through the induction of several cytokines, such as IL-6 and TNFα. This subsequently leads to apoptosis of melanocytes and further activation of innate immune cells [113].

There is additional evidence linking viral and bacterial infections to the development of vitiligo [114,115]. Since innate cells can recognize viral and bacterial components, commonly known as pathogen-associated molecular patterns (PAMPs), they can also serve as a pathway for their activation. Although several viral agents such as human immunodeficiency virus (HIV), herpes simplex viruses (HSV), varicella zoster virus (VZV), Epstein–Barr virus (EBV), and cytomegalovirus (CMV) have been investigated in context of vitiligo, their role in vitiligo remains unclear. Interestingly, recent study reported increased expression of cytosolic viral sensor MDA5 and anti-CMV IgM in patients with progressive vitiligo. MDA5 is encoded by IFIH1 which is recognized as one of the vitiligo susceptibility genes. Viral infection may activate MDA5 leading to the production of CXCL10 and CXCL16 by keratinocytes and recruitment of CD8+ T cells into the skin [105]. The impact of bacterial infections on vitiligo is currently uncertain although there are studies linking gut-skin dysbiosis to the appearance of vitiligo lesions and evidence of lesional skin dysbiosis compared to non-lesional skin in patients with vitiligo [115,116,117]. To summarize, although the evidence of the PAMPs activation pathway in vitiligo is still limited, it is certainly worth investigating given the link between other immune-mediated skin diseases and infectious triggers.

#### 3.2.2. Dendritic Cells (DCs)

DCs play a role in connecting oxidative stress with the adaptive immune response in vitiligo. They are antigen presenting cells (APC) responsible for T-cell activation, cytokine production, and immune response regulation. Following the recognition of DAMPs, such as HSP70i, DCs release high levels of IFN-α [118]. IFN-α induces the production of CXCL9 and CXCL10 by keratinocytes, thereby orchestrating the differentiation of T helper (Th) cells into the Th1 phenotype [119]. The role of plasmacytoid DCs (pDCs) as potent producers of IFN type I has been established in vitiligo. In active vitiligo lesions, PDCs expressing the HSP70 receptor, Lox-1, are primarily found near keratinocytes expressing HSP70. Furthermore, exogenous HSP70 can directly activate pDCs and induce IFN-α production, as demonstrated by the infiltrates of DCs and increased levels of IFN-α in the perilesional skin of patients with vitiligo [102,119]. However, a recent study found decreased levels of IFN-α in the circulation of patients with vitiligo, and increased levels of circulating DCs, IL-12, and TNF-α in patients with non-segmental vitiligo [120]. Another study demonstrated a relationship between the production of pro- and anti-inflammatory cytokines (IL-17A, IL-10, and IL-12p70) by DCs in both stable and active patients with vitiligo. The authors discovered that individuals with active vitiligo had higher levels of pro-inflammatory DCs (CD11b+CD11c+) in their peripheral blood mononuclear cells (PBMCs) and their skin than patients with stable vitiligo [63,121]. Interestingly, the levels of anti-inflammatory DC (CD11b+) and anti-inflammatory cytokine IL-10 were decreased in patients with active vitiligo, in contrast to an increased level of the pro-inflammatory cytokine IL-17A [121]. This latest data highlights the role of DCs in progression of vitiligo through modulation of pro- and anti-inflammatory cytokines.

#### 3.2.3. Natural Killer (NK) Cells and Innate Lymphoid Cells Type 1 (ILC-1)

Natural killer (NK) cells are involved in the early development of vitiligo as key initiators of the adaptive immune response [67]. NK cells, together with the innate lymphoid cells type 1 (ILC-1) are members of innate lymphocytes that form the first line of defense against infectious triggers and tumor cells [67,122,123]. In addition, NK cells possess a cytotoxic and regulatory function and are able to identify and destroy cells exposed to stress through activation receptors or limit the immune response through inhibitory receptors [123]. Previous studies have found elevated levels of circulating NK cells in patients with vitiligo; however, their role in the development of vitiligo was unclear, until recently [36,124]. Recent data indicate increase in basal expression of NK and ILC-1 cells in the perilesional skin and blood of patients with vitiligo [67]. In addition, NK cells receptors, including natural killer cell triggering receptor (NKTR), killer cell lectin-like receptor C1 (KLRC1), CCL20, and NK cell-related cytokines such as TNFα and IL-15, are also increased in perilesional skin in patients with vitiligo [125]. These data are consistent with the first analysis of the transcriptome of vitiligo skin genes, which showed increased expression of genes associated with the innate immune response, especially the function and activity of NK cells [101]. Furthermore, NK cells produce a wide range of cytokines and chemokines that aid in recruitment and activation of the adaptive immune cells [123]. In response to oxidative stress, as well as DAMP molecules such as HSP70 and HMBG1, NK and ILC-1 increase their production of IFN-γ in patients with vitiligo [67]. In addition, stressed NK and ILC-1 cells are able to induce keratinocyte and melanocyte production of chemokines CXCL9, CXCL10, CXCL11 and IFN-γ [67]. Therefore, it seems that NK cells, together with ILC-1, play a key role in the initial steps of vitiligo by modulating melanocyte and keratinocyte oxidative response resulting in production of IFN-γ and chemokines with consequent infiltration of CD8+ melanocyte-specific T lymphocytes.

### 3.3. Adaptive Immunity

#### 3.3.1. Cytotoxic CD8+ T cells

Vitiligo is a complex immune-mediated disease in which cytotoxic T lymphocytes target melanocytes, resulting in skin depigmentation. Although the immunopathogenesis of vitiligo remains unresolved, cytotoxic T-lymphocytes play a central role in the onset and clinical course of the disease. CD8+ T lymphocytes infiltrate vitiligo lesions, where they specifically target melanocytes [126,127,128,129]. Autoreactive CD8+ T lymphocytes produce interferon-gamma (IFN-γ), as demonstrated by its increased levels in vitiligo lesions [128]. IFN-γ-dependent mobilization of T cells to peripheral tissues involves the stimulation of chemokine secretion and expression of adhesion molecules on endothelial cells in skin lesions. Furthermore, IFN-γ stimulates the secretion of CXCL9 and CXCL10 by keratinocytes, which are responsible for guiding antigen-specific autoreactive CD8+ T lymphocytes into the skin and for inducing their effector functions [67,130]. Increased expression of the chemokines CXCL9 and CXCL10 and their associated CXCR3 receptor has been observed in vitiligo [65] (6). The levels of CXCL9 and CXCL10 correlate with clinical activity, whereas CXCL10 is linked to severity of the disease [65,130]. The accumulation of CD8+ T lymphocytes near epidermal melanocytes constitutes the effector phase of the anti-melanocyte response in vitiligo. Infiltrates of T lymphocytes, predominantly of the CD8+ phenotype, in addition to CD4+ T lymphocytes, were found in the lesions of patients with vitiligo, indicating an anti-melanocyte response mediated by CD8+ T lymphocytes [131]. Stimulation with melanocyte-specific antigens leads to the activation of CD8+ T lymphocytes, as demonstrated by the increased expression of cytotoxic markers such as granzyme-B, perforin, IFN-γ, and tumor necrosis factor-alpha (TNF-α, from tumor necrosis factor-alpha) [132]. Finally, CD8+ T lymphocytes produce increased levels of granzyme B, perforin, and IFN-γ, and highly express apoptosis stimulation fragment ligand (FasL) causing melanocyte death [68]. In addition to the aforementioned cytolytic molecules, recent data indicate another pathway of melanocyte apoptosis via chemokine-receptor-3 (CXCR3) isoform B CXCR3B. CXCR3B is found on the surface of melanocytes, and binding of its ligand, CXCL10, leads to melanocyte apoptosis. This apoptotic pathway is amplified in the presence of CD8+ lymphocytes [66,67].

#### 3.3.2. Regulatory T Cells

Regulatory T cells (Tregs) are CD4+ T cells crucial for providing peripheral tolerance to self-antigens by suppressing self-reactive CD8+ T cells. They inhibit the effector functions of T-cell directly by cell to cell contact or through the secretion of inhibitory cytokines [133]. Tregs are distinguished by the expression of CD4+, CD25+, and forkhead box P3 protein (FOXP3) [134,135]. The current literature on vitiligo places alterations in the number and function of Treg cells as important factors to the development of vitiligo. Previous research suggests an altered ratio of Treg cells in favor of CD8+ T cells, which may lead to sustained activation of anti-melanocyte CD8+ T cells [136,137]. In addition, Treg expression is reduced even beyond the borders of depigmentation in vitiligo skin, thus allowing CD8+ to attack melanocytes [138,139]. The lack of Tregs in vitiligo is caused by a decrease in the Treg homing receptor CCL22 in the skin of vitiligo patients [138,139]. Interestingly, by introducing CCL22 into the skin it was possible to cease depigmentation in a mouse model of vitiligo [138,139,140]. Tregs from active vitiligo lesions bear several functional defects, as evidenced by lower expression of immunosuppressive cytokines such as TGF-β and the transcription factor FoxP3 [135,137,141]. FoxP3 is important for its role in cytokine modulation as it increases the expression of immunosuppressive molecules such as CD25 and CTLA-4 while decreasing the expression of IL-2 and IL-4, which are required for T-cell activation [141]. However, more research is needed to determine the role of Tregs in vitiligo as well as therapeutic strategies based on modulating Tregs function.

#### 3.3.3. Tissue-Resident Memory T Cells

Tissue-resident memory T (TRM) cells comprise a diverse population of non-recirculating memory T cells important for assuring localized protection against reinfection with pathogens or foreign antigens [142,143,144]. Their role in many immune-mediated skin diseases, including vitiligo, has recently been apostrophized. The clinical course of vitiligo is marked by periods of stable disease, often complicated by periods of relapse in areas previously affected by vitiligo. Autoreactive skin TRM are thought to be responsible for vitiligo relapses in the event of different triggers. The population of TRM has already been demonstrated in the lesional and non-lesional skin of patients with vitiligo [145,146,147]. Skin TRM are characterized by the expression of CD69 (a C-type lectin), CD103 (integrin αE), and facultative expression of CD49a [144,145,148]. Interestingly, recent studies have revealed the presence of melanocyte-specific CD8+ TRM in the perilesional skin of patients with stable and active vitiligo [149]. This indicates their endurance in the skin following T-cell-driven inflammation and their ability to modulate future relapses or sustain depigmentation. The CD4+ TRM cells are present in the dermis, whereas melanocyte-specific CD8+ TRM are located in the epidermis of perilesional skin, near the melanocytes of patients with vitiligo [147,149]. CD8+ TRM are potent producers of IFN-γ and TNFα, which induce keratinocytes for CXCL9 and CXCL10 production and additional TRM influx into the skin. The recruitment of TRM cells to the skin is mediated by the CXCR3 receptor through its ligands, CXCL9 and CXCL10 [147,150,151]. CD49a has recently been identified as a marker of cytotoxic CD8+ TRM cells, which are potent producers of IFN-γ. However, their expression of key cytotoxic mediators, such as perforin and granzyme, relies on IL-15 production by keratinocytes and innate immune cells. In addition, CD8+ TRM express the C-type lectin-like receptor Natural Killer Group 2D (NKG2D) which is a marker of their activation [145,146,150]. Another interesting finding is the presence of melanocyte-specific but not activated TRM cells in the non-lesional skin of patients with vitiligo [146]. These cells could potentially lead to the emergence of new vitiligo lesions if appropriate triggers are present [147].

#### 3.3.4. The Role of B-Lymphocytes

In addition to T-cell mediated immunity, humoral pathways have been linked to the immunopathogenesis of vitiligo. B-cells are increased in epidermis of patients with vitiligo compared to healthy controls [152]. Additionally, there is evidence on a number of melanocyte-specific antibodies found in the sera of vitiligo patients which correlate with the disease’s activity. The presence of anti-melanocyte antibodies indicates a systemic immune activation in non-segmental vitiligo [14,153]. Furthermore, recent study revealed increased proportion of circulating memory B cells and decreased levels of naive B cells in active vitiligo, which is followed by an increase in circulating T follicular helper (cTfh) cells [154]. However, more research is needed to determine the precise function of B-cells and humoral mechanisms in vitiligo.

### 3.4. Main Cytokine Networks

The main cytokine networks explored in the pathogenesis of vitiligo are IFN-γ and TNF-α, in addition to IL-15 and Th17-related cytokines such as IL-17 and IL-23. The IFN-γ signalling pathway is well established in the pathogenesis of vitiligo, especially during the initiation of the disease. The major producers of IFN-γ are NK and ILC-1 cells, which are increased in the blood and non-lesional skin of vitiligo patients [67]. The main triggers leading to IFN-γ production are ROS, Hsp70i, and HMGB1, resulting in the increased expression of CXCR3B by melanocytes, as well as CXCL9 and CXCL10 by keratinocytes [67]. CXCL9 and CXCL10 are CXCR3 ligands that are important for the recruitment of adaptive immune cells to the skin and progression of vitiligo [155]. IFN-γ operates through the JAK/STAT pathway, resulting in the activation of JAK1-2 and STAT1 [59]. The JAK/STAT pathway consists of JAK1, JAK2, JAK3, and tyrosine kinase 2 (TYK2), which mediate cytokine signaling and induce innate and adaptive responses. Specifically, IFN-α activates JAK1/TYK2 and IFN-γ mediates its effects through JAK1/JAK2, IL-2, and IL-15 by initiating the JAK1/JAK3 pathway. The importance of the JAK pathway in vitiligo pathogenesis is evidenced by promising results achieved with JAK inhibitors in ongoing clinical trials. TNF-α, on the other hand, mediates its action through receptors TNFR1 or TNFR2, leading to the induction of mitogen-activated protein kinase (MAPK) and nuclear factor-kappa B (NF-kB) pathways [156]. Although it would be reasonable to propose TNF-α inhibitors for the treatment of vitiligo, the results of TNF-α inhibitors for treatment of vitiligo are not promising and even resulted in worsening of vitiligo [156]. Together, IFN-γ and TNF-α play a role in interfering with melanogenesis by directly decreasing the process of pigmentation. The combined role of these two cytokines was clearly evident in a recent study showing disruption of E-cadherin, resulting in the detachment of melanocytes from the basal layer of the epidermis in the presence of IFN-γ and TNF-α [97]. Another important pathway is the IL-15 cytokine pathway which has recently emerged in the pathogenesis of vitiligo, particularly through its role in the regulation of TRM [145,148]. IL-15 mediates its actions through the CD122 receptor subunit found on the surface of TRM. IL-15 is primarily produced by epidermal basal keratinocytes. Furthermore, IL-15R, which is essential for IL-15 transpresentation, is increasingly expressed by perilesional keratinocytes in patients with vitiligo [157]. Recent data linked the oxidative stress to an increased expression of IL-15 and IL-15Rα in the epidermis of vitiligo patients. In addition, IL-15 is able to intensify the cytotoxic potential of CD49a+ TRM cells, as well as the production of IFN-γ, the major driver of vitiligo [145]. The relevance of the IL-15 pathway is further evident from the promising results achieved through inhibition of IL-15 in a mouse model of vitiligo [146]. Another cytokine axis explored in the context of vitiligo is the IL-23/IL-17 axis [158]. Some in vitro studies have demonstrated IL-17 ability to shrink melanocytes, inhibit melanogenesis, and induce autophagy by impairing mitochondrial function via cytokines IL-1β, IL-6, and TNF-α [159]. Furthermore, there is evidence of an increase in Th17 lymphocytes, IL-17, and IL-23 levels in the serum and skin of vitiligo patients [160]. Recent studies, on the other hand, have found no evidence of elevated IL-17 levels in skin of patients with vitiligo when compared to healthy or psoriatic skin [147]. Furthermore, in a single-arm pilot study, the IL-17A inhibitor secukinumab failed to produce clinical results in the treatment of vitiligo and even worsened the condition in the majority of patients [161]. Therefore, the role of IL-17 pathway in the development of vitiligo remains to be elucidated.

## 4. Future Perspective of Vitiligo Treatment

The goal of vitiligo treatment is to slow the progression of the disease, to induce repigmentation and prevent relapses. Currently, non-targeted immunosuppressive therapies such as systemic and topical corticosteroids, topical immunomodulators, phototherapy, and surgical modalities are used to treat vitiligo. However, several targeted therapies are currently being investigated in light of new data on the immunopathogenesis of vitiligo. There are multiple clinical studies involving JAK/STAT inhibitors, as well as preclinical studies involving the IL-15 pathways, studies targeting the production of ROS, DAMPs and antioxidant pathways, or chemokine receptors, and stimulating melanocyte stem cells [59]. A pre-clinical study on a vitiligo swine model demonstrated that blocking one of the DAMPs associated with vitiligo, HSPi, could induce repigmentation of vitiligo lesions. Nonetheless, more research is required to confirm promising pre-clinical HSPi inhibition results [110]. Another intriguing target is IL-15 blockade, which has shown promising results in pre-clinical studies using a vitiligo mouse model. IL-15 is a key activator of TRM, which are responsible for vitiligo recurrence at previously affected sites. More research is needed, however, to determine the safety profile of the IL-15 blockade [146]. Chemokine receptor CXCR3 is an additional target for treating vitiligo with some good results in pre-clinical studies [162]. Pre-clinical studies with the aim of reintroducing Tregs to the skin by using CCL22 also demonstrated good efficacy [140]. However, the JAK/STAT pathway is undoubtedly the most promising target, with two drugs targeting this pathway currently in clinical trials, namely ruxolitinib and tofacitinib [163,164,165].

To summarize, despite significant progress in understanding vitiligo and developing targeted therapy, there is still room for discovering additional specific targets focused on stabilization and repigmentation of vitiligo.

## 5. Conclusions

Vitiligo is a complex chronic immune-mediated skin disease resulting in disappearance of melanocytes from the skin. The prevalence of vitiligo is 0.5–2% in general population, thus making it the most common depigmenting skin disease. Patients affected by vitiligo carry a significant psychological burden, therefore finding targeted treatment for vitiligo is of most importance. In recent years, significant progress has been made in understanding the immunopathogenesis of vitiligo. Latest research on vitiligo immunopathogenesis highlights the role of innate immune cells such as NK and ILC-1. The innate immunity cells link the oxidative stress to adaptive immunity, thus play a key role in initiation of the disease. Among the already established CD8+ lymphocytes, tissue resident T-cells have recently emerged as crucial immune cells capable of inducing relapse of vitiligo. Cytokine mediators such as IFN-γ and IL-15 are key cytokine drivers of vitiligo. IFN-γ operates through JAK-STAT pathway which is particularly relevant for development and maintenance of vitiligo, whereas IL-15 activates T-resident memory cells. Non-immune cells such as keratinocytes and fibroblasts also have a role in vitiligo, since complex interplay between immune cells and their cytokines leads to their activation. Additional investigations of these pathways may provide an opportunity for finding possible therapeutic targets, as there are currently no targeted biological drugs available for treatment of vitiligo.

## Data Availability

Data sharing not applicable.
